# Aberrant Salience and Disorganized Symptoms as Mediators of Psychosis

**DOI:** 10.3389/fpsyg.2022.878331

**Published:** 2022-04-12

**Authors:** Celia Ceballos-Munuera, Cristina Senín-Calderón, Sandra Fernández-León, Sandra Fuentes-Márquez, Juan Fco. Rodríguez-Testal

**Affiliations:** ^1^Personality, Evaluation and Psychological Treatment Department, University of Seville, Seville, Spain; ^2^Department of Psychology, University of Cádiz, Cádiz, Spain; ^3^Penitenciary Psychiatric Hospital, Seville, Spain; ^4^Clinical Mental Health Management Unit, Hospital Juan Ramón Jiménez, Huelva, Spain

**Keywords:** ideas of reference, preoccupation, psychosis, aberrant salience, psychopathology

## Abstract

**Introduction:**

Ideas of reference (IR) are frequent in psychopathology, mainly in psychotic disorders. The frequency of IR and preoccupation about them are related to the psychotic dimension, and to a lesser extent, to negative or emotional disorganized dimensions. Aberrant salience (AS), has been proposed as an indicator of the onset of psychosis, particularly of schizophrenia. This study analyzed the mediating role of AS, disorganized symptoms and preoccupation about IR in the relationship between IR and the psychotic dimension.

**Method:**

The sample consisted of 330 participants (116 university students and 214 clinically active patients), 62.4% of whom were women aged 18–79. The Referential Thinking Scale, the Aberrant Salience Inventory, and the Brief Psychiatric Rating Scale were administered.

**Results:**

Evidence of a partial mediation model showed that the relationships between IR and the psychotic dimension were mediated jointly by AS and the disorganized dimension, and preoccupation about IR no longer had a role. This relationship was significantly influenced by participant age. The variables in the model explained 54.16% of the variance.

**Conclusion:**

The model proposed enabled a set of vulnerabilities (unusual thought content) to be predicted that could lead to a high-risk general pathological state and proneness to psychosis in particular. These findings are discussed with regard to early detection and prevention of psychosis.

## Introduction

A large part of the effort devoted to the study of psychotic disorders has been directed at early identification of psychotic experiences and attenuated psychotic symptoms, thereby establishing rates of transition to psychosis, functioning profiles, and intervention designs (Colizzi et al., 2020; Fusar-Poli et al., 2020; Raballo et al., 2020).

In a longitudinal study, Addington et al. (2015) emphasized the importance of unusual thought content, particularly of suspicion and persecutory ideas. However, unusual thought content also shapes other very common important psychotic experiences, such as ideas of reference and aberrant salience (Miller et al., 2003; Yung et al., 2005).

Ideas of reference (IR) are a type of self-referential processing defined as self-attributions about what happens in the social environment (Senín-Calderón et al., 2017). They are considered one of the subclinical psychotic experiences grouped under the conceptual umbrella of schizotypy, which are relatively frequent in the general population (Lenzenweger et al., 1997; Rodríguez-Testal et al., 2019). Studies have suggested that IR are clinically important because their frequency, stability and severity are related to psychosis (Reininghaus et al., 2016; Marshall et al., 2019; Rodríguez-Testal et al., 2019; Cicero et al., 2020).

Research on IR has tried to establish its relationship with other symptoms and diagnoses, in addition to psychotic disorders, based on differences in their content (observation, communication, guilt or shame, communication media, causality, etc.) or their emotional effects (pleasant or unpleasant) (Wing et al., 1974; Lenzenweger et al., 1997; Startup and Startup, 2005; Cicero and Kerns, 2011; Senín-Calderón et al., 2014). Recently, (Wong et al., 2021) proposed considering IR according to their relationship with events in the setting which could bring them on (e.g., severe social events or a pandemic), between those that could be similarly interpreted by others, and those which are characteristic of people who experience them more strongly. They called the first IR attenuated and the second exclusive, characterizing a continuum of severity up to delusional intensity.

IR has been a basic symptom in proposals for Clinical High Risk assessment (Gross et al., 2008; Schultze-Lutter et al., 2010) as an attenuated psychotic symptom (Yung et al., 2005), as alterations in self-experience (Parnas et al., 2005), on a continuum leading up to delusional activity as delusional ideas of reference (Wong et al., 2012), and with regard to delusional ideas of persecution (Green et al., 2008).

Some IR parameters, such as the distress they cause, preoccupation about them, or specific content can be considered clinically significant (Green et al., 2008; Wong et al., 2012, 2021). Some initial data suggest that preoccupation about the presence of IR differentiates diagnostic categories more clearly, particularly those characterized by psychotic symptomatology (Senín-Calderón et al., 2016). Although some results suggest more importance of IR (Pelizza et al., 2019), there is an outstanding lack of studies on the role of parameters such as preoccupation about them, or the processes involved in these parameters.

According with van Os (2009), aberrant or anomalous salience (AS) has been proposed as a characteristic indicator of onset of psychotic disorders, particularly of schizophrenia. Kapur (2003), suggested that it consists of a motivational and attentional change or alteration toward stimuli from the setting, in such a way that neutral and irrelevant stimuli become abnormally salient. AS would then be the alteration of the natural motivational process toward novelty/reward attributed to the dopaminergic dysregulation traditionally linked by some authors to the onset of psychosis (Kapur et al., 2005; Raballo et al., 2019). From this perspective we might wonder whether the presence of IR, as a social cognition process, could be related to later increase in contextual ambiguity, perplexity, the still uncertain changes in meanings, which identify AS, and were classically called the trema stage (Conrad, 1958). Thus, AS may be a condition mediating the appearance of the first IR and later abnormal significance [the apophenia stage (Conrad, 1958), leading to the crystallization of delusion (Stanghellini et al., 2020)].

Therefore, AS could suggest a level of disorganization in cognitive functions, as observed in studies analyzing unusual thought content (Wilcox et al., 2014; Addington et al., 2015). In fact, Sass et al. (2018) allude to perceptual dysintegration, relating AS and other deficits that affect control and monitorization of cognitive processes. Thus, the emergence of AS could be related to more general disorganization and could be connected with other parameters of IR such as preoccupation about their content. This set of variables would be globally related to positive symptomatology.

Both IR (i.e., self-referencing) and AS can be included among the self-disorders (Mishara et al., 2016), although these cognitive processes may therefore appear at different moments in the development of psychosis. Self-disorders or anomalous subjective experiences (affectation of first-person experience, of its limits, its internal processes, etc.), are considered closely related to schizophrenia and AS, specifically, as a better predictor of psychosis than attenuated psychotic symptoms (Feyaerts et al., 2021). The IR would be an example of non-specific self-disorders included among the self-disorders as a trait (of schizotypy) (Lenzenweger et al., 1997), while processes like AS are a state related to schizophrenia (Blain et al., 2020).

**FIGURE 1:**
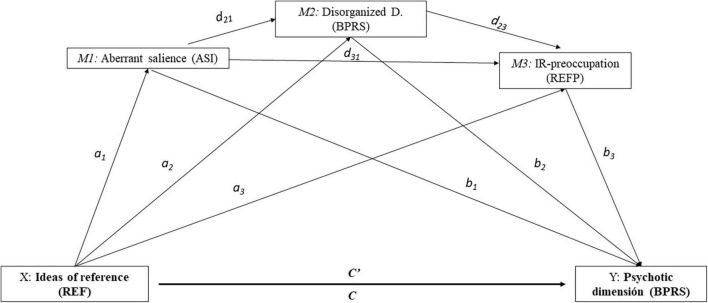
Conceptual model of the hypothesis proposed. a1, a2, a3 = effect of X on mediator variables (M1, M2, M3); b1 b2; b3 = effect of mediator variables on Y; c = Total effect of X on Y; c′ = direct effect of X on Y.

Identification of the mediators in any model proposed is essential to research. Mediation analysis enables researchers to go beyond mere relationships between variables by studying the possible effects of these relationships (MacKinnon et al., 2020). Although it is clear that a causal relationship between the variables cannot really be established, it does suggest a possible direction in the relationship between clinical variables and health (MacKinnon and Luecken, 2008). Another reason for studying mediators is the individual differences in the relationships between biological, psychological, behavioral and social factors. Mediation analyses can thereby suggest the development of efficient and effective intervention programs based on identification and later focus on the critical components of the program (O’Rourke and MacKinnon, 2018). In this sense and from a dimensional approach (van Os and Reininghaus, 2016), this study attempts to establish a series of relationships (not causal) among different indicators possibly related to the onset of psychosis. Thus, we propose a relationship between experiencing IR and the psychotic dimension of symptoms as a whole, and a tentative multiple mediation model to analyze this relationship. The hypothesis posed was that AS experiences, the disorganized dimension and preoccupation about IR sequentially mediate the relationship between the IR, as frequency of unusual thought content, and the appearance of a set of positive manifestations. The study’s hypotheses are organized in the theoretical model shown in Figure 1.

## Materials and Methods

### Participants

The study sample was made up of 330 participants (116 university students and 214 clinically active patients), aged 18–79, (*M* = 29.53; *SD* = 12.72), 62.4% of whom were women. The majority of participants were single (77.6%), 16.4% married or with a partner, 1.5% widowed, and 4.5% separated/divorced. In the group of students, 53.3% were women, the average age was 20.69 (*SD* = 3.45), and the average Social Class Index (Hollingshead, 1975) was 38.83 points (*SD* = 20.30; middle class; range 11–77); 4.3% were taking medication at the time of evaluation (anxiolytics) and 13.8% had a psychopathological history. In the group of patients, 46.7% were women, the average age was 34.32 (*SD* = 13.33), and the average Social Class Index was 46.41 points (*SD* = 22.40; low class; range 11–77). In this group, 67.8% were on medication at the time of the first interview, 77.5% had a psychopathological history and 35% had more than one diagnosis. Table 1 describes the sociodemographic characteristics of the participants and clinical diagnoses of the patients. The diagnoses were made by healthcare professionals (the patients’ clinical psychologists) with long clinical experience following the DSM-IV-TR classification (American Psychiatric Association [APA], 2000).

**TABLE 1:** Sample sociodemographic characteristics and clinical diagnoses.

	Students (*n* = 116)	Patients (*n* = 214)
Sex (% women)	53.3%	46.7%
Age (*M, SD*)	20.69 (3.45)	34.32 (13.33)
Age range (years)	19–47	18–79
Diagnoses (*n*)	–	Depressive D. = 42 Adjustment D. = 10 Anxiety D. = 52 Schizophrenia and other Psychotic D. = 65 Bipolar D. = 13 Personality D.^1^ = 19 Other^2^ = 13

*^1^Personality disorders (main diagnosis): Schizoid = 1; Paranoid = 3; Schizotypy = 5; Histrionic = 1; Borderline = 5; Dependence = 1; Unspecified personality D. = 3. ^2^Other: Dissociative = 1; Somatoform D. = 3; Eating behavior D. = 1; Sexual D. = 1; Addictive D. = 1; Neurodevelopment D. = 2.*

### Instruments

*Referential Thinking Scale* (REF, (Lenzenweger et al., 1997). Validated Spanish version by Rodríguez-Testal et al. (2019). It evaluates the interpretation of casual situations where the person may think others are staring at, laughing at or taking notice of him or her. It consists of 34 items with a “true/false” answer format. Preoccupation about IRs is scored by adding a Likert-type scale from 0 to 5 to each item, where 0 shows not preoccupied about IR at all, and 5 very much preoccupied (Senín-Calderón et al., 2016). This test has various factors (laughter/comments, attention/appearance, guilt/shame; songs/communication media, and reactions/changes), but in this study, only the sum of its items in a global REF score was used, and for preoccupation (REFP) when IR was found to be present. The Spanish validation found adequate psychometric properties with ordinal α for the total score that varied from 0.94 (adolescents) to 0.97 (patients and adult general population). Internal consistency for this study had a Cronbach’s α = 0.88 for IR frequency, and 0.92 for preoccupation.

*Aberrant Salience Inventory* (ASI, (Cicero et al., 2010), validated in Spanish by Fernández-León et al. (2019). This scale was used to measure proneness to psychosis and evaluate the assignment of significance to usually irrelevant internal and external stimuli. It has 29 items with a dichotomous (true/false) answer format with five dimensions: Heightened cognition, impending understanding, heightened emotionality, increased significance, senses sharpening. The total score is found by adding up the items answered affirmatively. The authors reported a Cronbach’s *a* = 0.89 for the complete scale and evidence of validity compared to other measures of proneness to psychosis. The Spanish validation had adequate internal consistency (Cronbach’s α = 0.95) and evidence of validity compared to other measures of positive and negative symptoms. In this study, the Cronbach’s *a* = 0.90.

*Brief Psychiatric Rating Scale* (BPRS; Lukoff et al., 1986). Spanish validation by Peralta Martín and Cuesta Zorita (1994). This is an other-report scale with 24 items that evaluate positive (psychotic and disorganized) and negative emotional symptoms. The items are rated from 1 (absence of psychopathology) to 7 (extreme psychopathology). The 18-item Spanish version had a Cronbach’s *a* reliability of 0.59 to 0.70, and test-retest reliability *r* = 0.70. This study used only the psychotic dimension (Items 6. Suspiciousness, 7. Delusions, 8. Grandiosity, 9. Hallucinations) and the disorganized dimension (Items 11. Conceptual disorganization, 12. Excitement, 21. Bizarre behavior, 22. Elation, 23. Hyperactivity, 24. Distractedness). Internal consistency in this study was Cronbach’s *a* = 0.85 for the psychotic dimension and Cronbach’s *a* = 0.82 for the disorganized dimension.

### Procedure

All participants were informed of the objectives of the study and gave their written consent for participation. The sample was collected by incidental sampling. The participants from the general population were university students who voluntarily agreed to fill in the study tests in class, and later were evaluated in an individual interview. These students received points toward their grade for participating at both evaluation times. The group of patients was collected by incidental sampling at a private psychology clinic and various public hospitals. They filled in the tests in writing, and at another time, were individually interviewed. This study followed the precepts of the Declaration of Helsinki and was approved by the Ethics Committee of the Junta de Andalucía [Andalusian Government] (PI 010/16).

### Data Analysis

All data analyses were performed with SPSS.19 software. The theoretical mediation model proposed was tested following the recommendations of MacKinnon et al. (2004), using multiple mediation analysis with three mediators (AS, disorganized dimension and preoccupation about IR) using the PROCESS macro v3.5 (Hayes, 2017; Model 6) with bootstrapping. The coefficients were estimated from 10000 bootstrap samples and all analyses were accepted with a level of significance of *p* < 0.05.

## Results

### Descriptive Analyses

Before applying the mediation model, the relationships between the variables to be entered were tested. The results, in Table 2, show significant positive association between all the variables in the study. As observed in Table 2, differences were found between the ages of patients and non-patients [*t*_(261_._626)_ = 14.10; *p* < 0.001], so this variable was entered in the mediation model as a covariate.

**TABLE 2:** Means, standard deviations, and Spearman correlations between study variables.

	1.	2.	3.	4.	5.	6.	*M*	*D.T*
1. REF	–	0.472**	0.563**	0.368**	0.876**	0.08**	5.466	5.456
2. BPRS- Psy		–	0.545**	0.712**	0.408**	0.434**	1.860	1.199
3. ASI			−	0.508**	0.535**	0.157**	10.888	6.767
4. BPRS-Dis				−	0.285**	0.482**	1.432	0.544
5. REFP					−	-0.049*	10.350	16.033
6. Age						–	29.53	12.72

N = 330; 1 = IR; 2 = Psychotic dimension; 3 = Aberrant salience; 4 = Disorganized dimension; 5 = Preoccupation about IR. *p < 0.05; **p < 0.01. (bilateral).

### Mediation Analysis

According to Baron and Kenny (1986), four conditions must be met to establish a theoretical mediation model: The IV or predictor, must be related to the DV or criterion; the predictor must be related to the mediator variables; these mediators have to be related to the criterion variable once the effect of the IV is controlled for. Finally, the effect of the predictor variable on the criterion variable must be reduced when the effect of the mediator variables is controlled for. The results of the multiple mediation analysis are shown in Table 3.

**TABLE 3:** Total, direct and indirect effects based on 10,000 bootstrap samples.

	*B*	*SE*	*t*	*p*	CI 95% (upper and lower)
Total effect (X→Y)	0.087	0.009	9.902	0.001	[0.070, 0.104]
Direct (X→Y) effect	0.027	0.013	2.055	0.041	[0.001, 0.053]
Age (covariate)	0.009	0.004	2.226	0.027	[0.001, 0.016]
Indirect effects					
−1: X→M1→Y	0.027	0.008			[0.015, 0.044]
−2: X→M1→M2→Y	0.016	0.005			[0.008, 0.026]
−3: X→M1→M3→Y	0.001	0.001			[−0.001, 0.001] (ns)
−4: X→M1→M2→M3→Y	0.001	0.001			[−0.001, 0.000] (*ns*)
−5: X→M2→Y	0.015	0.006			[0.005, 0.028]
−6: X→M2→M3→Y	0.001	0.001			[−0.001, 0.000] (ns)
−7: X→M3→Y	0.002	0.013			[−0.027, 0.026] (ns)

*X = IR; Y = psychotic D; M1 = SA; M2 = Disorganized D.; M3 = Preoccupation about IR; ns = not statistically significant (as shown by a bootstrap confidence interval including 0); CI = Confidence interval. The coefficients are unstandardized.*

**FIGURE 2:**
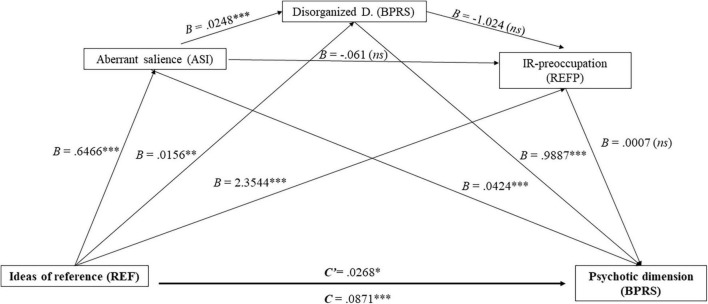
Mediation model proposed and tested; c′ = direct effect of X; c = total effect of X on Y; on Y; ^**^p < 0.01; ^***^p < 0.001; *ns* = not statistically significant. Coefficients shown are unstandardized.

The presence of IR had a significant total effect on the psychotic dimension. When AS experiences, disorganization and preoccupation about IR were added to the model, the size of the beta coefficient decreased, although still remaining significant. The total indirect effect was statistically significant (*B* = 0.060, IC 95% [0.031,0.090]). The results suggest that AS, disorganization and preoccupation about IR, partially mediated the relationship between presence of IR and the psychotic dimension. As shown in Table 3, the indirect effects of AS and disorganization on the psychotic dimension were significant, in interaction as well as alone. Age was significant (*B* = 0.009, IC 95% [0.001,0.016]) in the relationship between IR and the psychotic dimension, effect which was controlled for. The overall model represented 54.16% of the variance in the total score of the psychotic dimension, *R*^2^ = 0.54, *F* (5, 324) = 76.56, *p* < 0.001, with a large effect size (Cohen’s *f*^2^ = 1.18). The final model is shown in Figure 2.

## Discussion

In spite of the strong presence of IR in human nature itself, and of being a type of mental activity historically characterized in both evolutionary development and the clinical context, there are few studies directed specifically at this cognitive process, and its mention has been relegated mainly to the delusional form (Senín-Calderón et al., 2014).

Similarly, AS, traditionally related to psychosis because it represents the emergence of alterations of the self (Henriksen and Parnas, 2019), can also be objectivized on the continuum of human experience (Fyfe et al., 2008), possibly because of its relationship with other processes as common as depersonalization or absorption (Sass et al., 2018; Fernández-León et al., 2020).

This study attempted to explore these two unusual thought contents, IR and AS, by examining their possible dependence, connection with disorganization processes and finally, their possible relationship with the psychotic dimension. In the mediation model proposed, the hypothesis is that AS experiences, product of the frequency of IR, lead to an increase in disorganization indicators, which in turn, could be associated with greater preoccupation about the presence of IR, and finally, with more symptoms characteristic of the psychotic dimension. Evidence was found for a mediation model, according to which the relationships between IR and psychotic dimension symptoms were both mediated by AS and the disorganized dimension, and preoccupation about IR no longer had a role.

The findings of this study are consistent with others that have found a statistically significant positive relationship between the referential thinking scale (REF) and the ASI (Chun et al., 2019; Rodríguez-Testal et al., 2019), as well as the CAPE scale of proneness to psychosis (Fonseca-Pedrero et al., 2012), so it may be said that both tests are clearly related to psychosis, although the REF scale seems to be somewhat more specifically so, at least in the general population (Senín-Calderón et al., 2014). When these scales were used to evaluate IR and AS, it was demonstrated that the latter must participate in development of IR, although they are characterized in a wider context of psychotic disorganization (Cicero et al., 2010). It was also demonstrated that the assessment of preoccupation about IR is significantly related to measures of psychotic symptomatology, and also with emotional components that indicate clinically significant distress (Senín-Calderón et al., 2016).

The tradition of the continuum of unusual thought content, such as IR, joins the German tradition of Robert Gaupp (Mishara and Fusar-Poli, 2013) and Ernest Kretschmer (Rodríguez-Testal et al., 2016), who described aspects related to personality, which would place sensitivity (asthenia) at the base of sensitive delusion of reference, precipitated by life events (Kretschmer and Strauss, 1952). The excellent recent study by Wong et al. (2021) suggests that, although the IR are precipitated by a context, the exclusiveness with which they are experienced must be considered. These authors emphasize the mediating role of rumination between events experienced and the severity of the IR. Rumination about the event probably has this role. In brief, IR can occur during general cognitive functioning, but requires the mediation of rumination to become exclusive to psychosis. In this study, the mediating role of preoccupation, but specifically about the IR themselves, was not verified, although it was significantly correlated with all the measures. It is therefore possible that its role is rather of moderator, as proposed in other studies, although with negative affect as the predictor (Ludwig et al., 2020).

The reconsideration of alterations of the self as diagnostic criteria for schizophrenia (ICD-11) (World Health Organization [Who]., 2018) attributes extraordinary importance to AS. However, some indications suggest that this indicator of disorganization of the self is not exclusive to schizophrenia (Neumann et al., 2020), although it may be more prominent, continuous, even a trait of this disorder (Blain et al., 2020). Possibly, as suggested by Feyaerts et al. (2021), most specificity of these alterations of the self occurs when processes such as hyperreflexivity (exaggerated self-consciousness) and diminished self-presence (diminished intensity or vitality of oneself) emerge.

This study did not intend to analyze the different diagnostic categories or verify whether the large group of participants diagnosed with active schizophrenia were characterized to a greater extent by AS. Therefore, one limitation of this study is precisely that it assumed a continuum perspective, including from participants from the general population to different diagnoses, with and without psychotic symptoms. However, we think that this perspective enables the processes in its relationship to be understood without considering diagnostic differences, since the interview took place at a different time from when the tests were filled out. Another limitation to be mentioned is its cross-sectional design, and incidental recruitment of the sample, so results cannot be generalized. However, the sequence of the mediation model could make it possible to explore the relationship of relevant variables and an approach to causality (Hayes and Preacher, 2014), although understood as strictly provisional. Some very important variables for discussing psychosis are also lacking, from negative symptoms to consideration of high negative affect and low positive affect, or facets related to upbringing and stressful situations (Olvet et al., 2015; Fernández-León et al., 2020; Healy et al., 2020).

Summarizing, a model and relationships between variables has been proposed in a sequence that could enable an approach to psychosis that takes unusual thought content as a starting point. This proposal enables vulnerabilities to be explored, which due to their onset and stability in process severity, may be useful indicators in the early detection of the psychosis for prevention or early intervention. The results followed the perspective that considers schizotypy a latent variable of personality organization, represented by a subtle, subclinical psychotic phenomenology (e.g., perceptive aberrations or ideas of reference) which could lead to a variety of phenotypical results related to schizophrenia (Lenzenweger, 2018). Unlike Meyer et al. (2021), these characteristics of schizotypy (trait, such as ideas of reference) could be the antecedent of state processes, such as aberrant salience. According to these authors, it is possible for impaired self-processing to appear. Current studies have attempted to design a more meticulous evaluation of the high-risk groups, forming more homogeneous subgroups stratified by variables (Carrión et al., 2017). In view of the mediator role found in this study, some expressions of self-disorders, particularly aberrant salience, would make a more detailed analysis of the psychosis prodromes possible. This study also supports the need for design and validation of reliable instruments for identification and detection of referential thought (ideas of reference) in clinical and general populations, a project on which we are now working on. This proposal would enable exploration of vulnerabilities, which due to their onset and stability in the severity of the process, may be useful as indicators to be kept in mind for early detection of the psychotic process for prevention or early intervention.

## Data Availability Statement

The raw data supporting the conclusions of this article will be made available by the authors, without undue reservation.

## Ethics Statement

The studies involving human participants were reviewed and approved by Ethics Committee of the Junta de Andalucía (Andalusian 156 Government) (PI 010/16). The patients/participants provided their written informed consent to participate in this study.

## Author Contributions

All authors listed have made a substantial, direct, and intellectual contribution to the work, and approved it for publication.

## Conflict of Interest

The authors declare that the research was conducted in the absence of any commercial or financial relationships that could be construed as a potential conflict of interest.

## Publisher’s Note

All claims expressed in this article are solely those of the authors and do not necessarily represent those of their affiliated organizations, or those of the publisher, the editors and the reviewers. Any product that may be evaluated in this article, or claim that may be made by its manufacturer, is not guaranteed or endorsed by the publisher.
